# A risk-based model for unplanned cesarean delivery following induction of labor in term hypertensive nulliparas

**DOI:** 10.1007/s00404-026-08445-9

**Published:** 2026-04-24

**Authors:** Avihu Krieger, Michal Axelrod, Baha Sibai, Shalom Mazaki-Tovi, Michal Fishel Bartal

**Affiliations:** 1 The Department of Obstetrics and Gynecology, The Chaim Sheba Medical Center, Ramat-Gan, Israel; 2 The School of Medicine, Tel Aviv University, Tel-Aviv, Israel; 3 The University of Texas Health Science Center at Houston, Houston, TX USA

**Keywords:** Hypertensive disorders of pregnancy, Prediction, Risk factors, Counseling

## Abstract

**Purpose:**

To develop a practical risk-stratification framework for unplanned cesarean delivery (CD) among term nulliparous individuals with hypertensive disorders of pregnancy (HDP) undergoing induction of labor (IOL).

**Methods:**

This was a retrospective cohort study at a single tertiary care center (January 2010-March 2025) of nulliparous individuals with singleton gestations diagnosed with HDP undergoing IOL at ≥ 37 + 0 weeks. We excluded multiple gestations, major fetal anomalies, planned CD, or intrauterine fetal death. We included demographic and pregnancy characteristics available prior to induction and evaluated association with unplanned CD. Stepwise backward logistic regression was used to build a model for identifying independent predictors of unplanned CD. Sensitivity, specificity, and likelihood ratios (LR) were calculated.

**Results:**

Among 1,326 eligible individuals, 347 (26.2%) underwent unplanned CD. Independent predictors of CD were age > 35 years (adjusted odds ratio [aOR] 1.97, 95% CI 1.45–2.66), body mass index ≥ 30 kg/m^2^ (aOR 2.07, 95% CI 1.58–2.70), HDP with severe features (aOR 1.71, 95% CI 1.17–2.49), thrombocytopenia (aOR 2.66, 95% CI 1.17–6.06), and need for cervical ripening (aOR 1.63, 95% CI 1.23–2.16). Cesarean risk increased stepwise with accumulation of risk factors: 28.4% with ≥ 1 factor, 36.7% with ≥ 2, 44.7% with ≥ 3, and 64.7% with ≥ 4. The presence of ≥ 4 factors yielded a positive LR of 5.17 (95% CI 1.92–13.99).

**Conclusion:**

In term nulliparous individuals with HDP undergoing induction, approximately one in four require CD. A simple model based on five routinely available pre-induction factors enables individualized counseling and shared decision-making at the bedside.

## What does this study add to the clinical work

**Table:** 

This study provides a simple, bedside risk-stratification model to estimate the likelihood of unplanned cesarean delivery among term hypertensive nulliparous individuals undergoing induction of labor. Using five routinely available pre-induction factors, clinicians can offer personalized, data-driven counseling to support shared decision-making and expectation setting before induction.	

## Introduction

Hypertensive disorders of pregnancy (HDP) are among the most frequent medical complications of pregnancy, affecting approximately 5–10% of pregnancies [1–3], and remain a leading cause of both maternal and perinatal morbidity and mortality [4]. For individuals at term, induction of labor (IOL) is a common management strategy [5], supported by professional guidelines [6] demonstrating favorable maternal outcomes without an increase in adverse neonatal events [7].

In the general obstetric population, several studies have shown that cesarean delivery (CD) following a failed induction is associated with higher maternal morbidity compared with planned CD, including increased rates of adverse outcomes such as endometritis and postpartum hemorrhage (PPH) [8, 9]. Consequently, accurate counseling regarding the likelihood of successful IOL and careful patient selection are essential components of clinical decision-making, particularly in identifying those for whom a planned CD may be the preferable option.

Despite the high prevalence of IOL in individuals with HDP, decisions regarding mode of delivery are still largely based on routine obstetric considerations [6]. Existing evidence on IOL in this group is primarily derived from heterogeneous cohorts that combine term and preterm gestations, as well as nulliparous and multiparous individuals [10], which may limit its direct applicability to nulliparous individuals at term. Large, population-based datasets can help address this gap by enabling the identification of robust, clinically relevant predictors in this specific high-risk group.

Therefore, the objective of our study was to identify risk factors associated with unplanned CD in nulliparous individuals presenting with HDP at term and to develop a clinical prediction model for informed decision making.

## Materials and methods

This was a retrospective cohort study conducted at a single tertiary medical center of all nulliparous individuals with singleton pregnancies diagnosed with HDP undergoing IOL at term (≥ 37 + 0 weeks of gestation) between January 2010 and March 2025. We excluded those with multiple gestations, major fetal anomalies, planned CD without trial of labor or intrauterine fetal death prior to initiation of induction.

The institution maintains a comprehensive perinatal database integrated with the electronic medical record (EMR), which contains detailed demographic, obstetric, delivery, and neonatal information. All deliveries during the study period were identified and extracted from the database. We identified our cohort using documentation codes for HDP including gestational hypertension, preeclampsia without severe features, preeclampsia with severe features, eclampsia, HELLP syndrome, or superimposed preeclampsia. For individuals assigned more than one HDP diagnosis during pregnancy, the more severe diagnosis was used for classification.

Gestational hypertension was defined as blood pressure elevation (systolic blood pressure ≥ 140 mmHg or diastolic blood pressure ≥ 90 mmHg) on two occasions at least 4 h apart in an individual with previously normal blood pressure in the absence of proteinuria or systemic findings as defined below. Preeclampsia without severe features was defined as gestational hypertension and proteinuria (≥ 300 mg per 24-h urine collection or protein/creatinine ratio ≥ 0.3 mg/dL). In the absence of proteinuria, preeclampsia was defined as new-onset hypertension with new systemic findings including any of the following: thrombocytopenia (platelet < 100 000/μl), renal insufficiency (serum creatinine > 1.1 mg/dL or a doubling of the serum creatinine concentration in the absence of other renal disease), impaired liver function (elevated liver transaminase to twice normal concentration), pulmonary edema, eclampsia, or cerebral or visual symptoms. Individuals were also considered to have preeclampsia with severe features if they had elevated blood pressure (systolic blood pressure 160 mmHg or diastolic blood pressure ≥ 110 mmHg) and proteinuria or new systemic findings as described previously.

Data was retrieved from electronic health records. Maternal variables included age, body mass index (BMI, calculated as weight in kilograms divided by height in meters squared), and smoking status. Pregnancy and delivery characteristics included mode of conception, HDP type and severity (with severe HDP defined as severe preeclampsia, eclampsia, HELLP syndrome, or superimposed preeclampsia with severe features), thrombocytopenia (defined as platelet count < 100 × 10⁹/L), elevated liver enzymes (AST or ALT > 2 times the upper limit of normal), administration of magnesium sulfate for maternal seizure prophylaxis at any time during pregnancy or delivery, and the need for cervical ripening, defined as administration of prostaglandins, balloon catheter insertion, or other unspecified ripening methods prior to oxytocin augmentation. This variable was used as a pragmatic surrogate for unfavorable cervical status, as detailed Bishop score components were not uniformly available in the dataset.

The primary outcome was mode of delivery, defined as vaginal delivery (successful induction) or CD following induction (unplanned CD). Cesarean deliveries were further categorized by indication as failure to progress, non-reassuring fetal status, or other less common causes. Operative delivery (vacuum or forceps assisted) was included in the vaginal delivery classification.

Secondary outcomes included maternal and neonatal complications. Maternal outcomes comprised of third-or fourth-degree perineal laceration, PPH (defined as estimated blood loss > 1000 mL within 24 h of delivery or requiring intervention), blood transfusion, and a composite adverse maternal outcome consisting of endometritis, hysterectomy, chorioamnionitis, uterine rupture, peripartum fever, blood transfusion and PPH.

Neonatal outcomes included birth weight, Apgar score < 7 at 5 min, and admission to the neonatal intensive care unit.

We estimated the required sample size for developing a multivariable logistic regression model to identify predictors of unplanned CD with at least 10–20 outcome events per predictor variable to reduce overfitting and support model stability. The anticipated CD rate used was derived from our hospital data which is similar to the general population. Assuming an unplanned CD rate of 30% and planning to include up to 10 candidate predictors, we estimated a required sample size between 334 and 667 participants to achieve 100–200 events. Our final cohort included 1,326 participants, exceeding the minimum requirement. This larger sample size strengthens the validity and generalizability of the model.

Baseline characteristics were summarized using descriptive statistics. Categorical variables were compared using the χ^2^ or Fisher’s exact test and reported as counts with percentages. Continuous variables were compared using the t-test or Mann–Whitney U test, as appropriate, and reported as mean ± standard deviation (SD) or median with interquartile range (IQR). Continuous variables such as maternal age and BMI were initially evaluated as continuous predictors and subsequently dichotomized for clinical interpretability and bedside applicability using commonly accepted thresholds (age > 35 years and BMI ≥ 30 kg/m^2^).

Univariable logistic regression was first performed to assess associations between each predictor and the risk of unplanned CD. Variables that were significant on univariable analysis (P < 0.05) were subsequently entered into a multivariable logistic regression model to identify independent predictors of CD following IOL. A backward stepwise approach was used to derive a parsimonious model; however, variable selection was not based solely on statistical criteria. Clinically relevant variables were prioritized and retained based on both statistical significance and clinical plausibility to enhance interpretability and applicability. Adjustment was performed formaternal age, BMI, severe HDP, thrombocytopenia and need for cervical ripening which were included in the final model.

.

BMI data was available for 908 of 979 (93%) individuals in the vaginal delivery group and 325 of 347 (94%) in the CD group; percentages for this variable were calculated accordingly. All other variables were complete for the full analytic cohort.

Magnesium sulfate and antihypertensive treatment were excluded from the model owing to collinearity with severe HDP. Results were expressed as adjusted odds ratios (aORs) with 95% confidence intervals (CIs). The discriminative ability of the multivariable model was assessed using the area under the receiver operating characteristic curve (AUC). To evaluate the cumulative effect of multiple risk factors, we constructed a risk stratification model by categorizing individuals according to the number of predictors present (≥ 1, ≥ 2, ≥ 3, or ≥ 4). For each threshold, sensitivity, specificity, and LR for CD were calculated. In addition, the rate of unplanned CD (%) was described for each risk group. Dose–response relationships were examined by plotting the proportion of unplanned CD across increasing numbers of risk factors.

A *P* value < 0.05 was considered statistically significant. Analyses were performed using SPSS software, version 27 (IBM Corp., Armonk, NY).

The study protocol was approved by the Institutional Review Board of Sheba Medical Center (Approval No. 2250–25-SMC). Given the retrospective design and use of de-identified data, the requirement for informed consent was waived.

This study was conducted and reported in accordance with the Strengthening the Reporting of Observational Studies in Epidemiology (STROBE) guidelines for cohort studies.

## Results

**Fig. 1:**
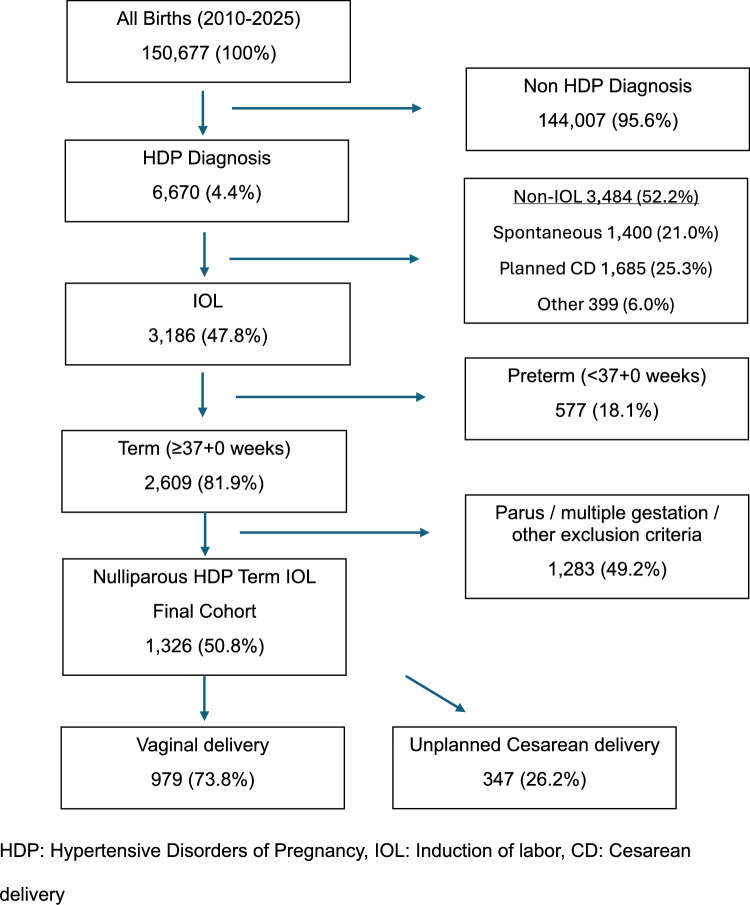
Study cohort selection and induction outcomes. Flow diagram depicting identification of term nulliparous individuals with hypertensive disorders of pregnancy undergoing induction of labor and final mode of delivery. HDP: Hypertensive Disorders of Pregnancy, IOL: Induction of labor, CD: Cesarean delivery

Of the 150,677 deliveries during the study period, 6,670 (4.4%) individuals were diagnosed with HDP. Among them, 3,186 (47.8%) underwent IOL, of those 2,609 at term. Following exclusion criteria, 1,326 nulliparous individuals with singleton pregnancies met inclusion criteria; 979 (73.8%) delivered vaginally, and 347 (26.2%) had an unplanned CD (Fig. 1).

Individuals undergoing an unplanned CD were older (32.2 vs 29.9 years, *p* < 0.001), had a higher BMI (31.6 vs 29.4 kg/m^2^, *p* < 0.001), and had a higher rate of smoking (6.1% vs 3.3%, *p* = 0.02) compared to those with successful vaginal delivery. Furthermore, the rate of conception via assisted reproductive technology (ART) (21.6% vs 13.4%, *p* < 0.001), HDP with severe features (16.7% vs 10.7%, *p* = 0.004), thrombocytopenia (3.5% vs 1.5%, *p* = 0.03), magnesium sulfate prophylaxis (15.0% vs 8.9%, *p* = 0.001), and need for cervical ripening (70.9% vs 59.5%, *p* < 0.001) were also more frequent among those who had an unplanned CD compared to successful vaginal delivery. (Table 1).

**Table 1:** Baseline maternal and pregnancy characteristics of term nulliparous individuals with hypertensive disorders of pregnancy undergoing induction of labor, by mode of delivery

Characteristics	Vaginal delivery (*n* = 979)	Unplanned Cesarean delivery (*n* = 347)	*p*-value
**Demographic and clinical characteristics**			
Age	29.9 ± 5.2	32.2 ± 5.2	< 0.001
> 35 years	164 (16.8)	100 (28.8)	< 0.001
BMI	29.4 (26.4–33.3)	31.6 (28.0–35.1)	< 0.001
≥ 30 kg/m^2a^	401 (44.1)	200 (61.5)	< 0.001
Smoking	32 (3.3)	21 (6.1)	0.02
**Pregnancy characteristics**			
ART	131 (13.4)	75 (21.6)	< 0.001
HDP with severe features^b^	105 (10.7)	58 (16.7)	0.004
Thrombocytopenia^c^	15 (1.5)	12 (3.5)	0.03
Elevated liver enzymes^**d**^	31 (3.2)	11 (3.2)	0.99
Oligohydramnios	63 (6.4)	20 (5.8)	0.66
Magnesium sulfate prophylaxis^e^	87 (8.9)	52 (15.0)	0.001
Cervical ripening^f^	583 (59.5)	246 (70.9)	< 0.001

*BMI* Body Mass Index, *ART* Assisted Reproductive Technology, *HDP* Hypertensive Disorders of Pregnancy, *CD* Cesarean delivery, *PPH*, Post Partum Hemorrhage

Data are presented as mean ± standard deviation, median (interquartile range), or N (%)

^a^BMI data was available for 908 of 979 individuals in the vaginal delivery group and 325 of 347 in the cesarean delivery group. Percentages are calculated accordingly

^**b**^Defined as any of the following: severe preeclampsia, eclampsia, HELLP syndrome or superimposed preeclampsia with severe features

^**c**^Defined as platelet count < 100 × 10⁹/L

^**d**^Defined as aspartate aminotransferase (AST) or alanine aminotransferase (ALT) > 2 times upper limit of normal

^e^Treatment was highly correlated with severe HDP and therefore excluded from the final model to avoid multicollinearity

^f^Defined as the use of a balloon catheter, prostaglandins, or unspecified cervical ripening methods. individuals not receiving ripening underwent augmentation only

Indications for unplanned CD included failure to progress (40.0%), non-reassuring fetal heart rate (39.1%), and other causes (20.7%). Individuals who had an unplanned CD had a lower rate of PPH compared to vaginal delivery (1.4% vs 4.6%, *p* = 0.01), with no difference in the overall composite maternal adverse outcome (15.9% vs 15.5%, *p* = 0.89). Neonatal birth weight was slightly higher in the CD group (3140 vs 3055 g, *p* = 0.03), and 5-min Apgar score < 7 were more frequent (1.2% vs 0%, *p* = 0.005) compared to vaginal delivery (Table 2).

**Table 2:** Delivery, Maternal and neonatal outcomes among term nulliparous individuals with hypertensive disorders of pregnancy undergoing induction of labor, by mode of delivery

Characteristics	Vaginal delivery (*n* = 979)	Cesarean delivery (*n* = 347)	*p*-value
**Delivery Outcomes**			
Gestational age at delivery	39.1 (38.0–40.1)	39.1 (38.0–40.2)	0.42
CD indication			
Failure to progress^**a**^		139 (40.1)	
Fetal distress^b^		136 (39.2)	
Other^c^		72 (20.7)	
Operative vaginal	171 (17.5)		
**Maternal Outcomes**			
3rd/4th degree perineal tear	21 (2.1)		
Composite maternal outcome^d^	152 (15.5)	55 (15.9)	0.89
Blood transfusion^e^	29 (3.0)	11 (3.2)	0.85
Endometritis	4 (0.4)	1 (0.3)	0.75
Postpartum infection^f^	24 (2.4)	12 (3.5)	0.32
Readmission	39 (4.0)	17 (4.9)	0.47
PPH	45 (4.6)	5 (1.4)	0.01
**Neonatal Outcomes**			
Weight, grams	3055 (2775–3380)	3140 (2790–3520)	0.03
Apgar score at 5 min below 7	0 (0.0)	4 (1.2)	0.005
NICU admission	1 (0.1)	3 (0.9)	0.06

*CD* Cesarean Delivery, *PPH* Post Partum Hemorrhage, *NICU* Neonatal Intensive Care Unit

Data are presented as mean ± standard deviation, median (interquartile range), or N (%)

^a^Includes arrest of dilation, failed induction, dysfunctional labor, and suspected cephalopelvic disproportion

^b^Includes non-reassuring Fetal heart rate

^c^Includes suspected placental abruption, maternal request, and other less common indications

^d^Includes Endometritis, Hysterectomy, Chorioamnionitis, Uterine rupture, Peripartum fever, blood transfusion and PPH

^e^Within 21 days of delivery

^f^Includes endometritis, mastitis, urinary tract infection, wound infection, surgical site infection, vaginitis, folliculitis, pneumonia, gastroenteritis, erysipelas, sepsis, and bacteremia occurring within 60 days Postpartum

On multivariable regression, age > 35 years (aOR 1.97, 95% CI 1.45–2.66), BMI ≥ 30 kg/m^2^ (aOR 2.07, 95% CI 1.58–2.70), severe HDP (aOR 1.71, 95% CI 1.17–2.49), thrombocytopenia (aOR 2.66, 95% CI 1.17–6.06), and need for cervical ripening (aOR 1.63, 95% CI 1.23–2.16) were independently associated with unplanned CD (Table 3). Using the regression model, we generated a receiver operating characteristic (ROC) curve, with an AUC of 0.66 (95% CI 0.63–0.69).

**Table 3:** Regression analysis of factors associated with cesarean delivery after induction in nulliparous individuals with singleton pregnancies at 37 weeks and beyond with hypertensive disorders of pregnancy

Characteristics	Vaginal delivery (*n* = 979)	Cesarean delivery (*n* = 347)	*p*-Value	aOR^#^ (95% CI)
Age > 35 Years	164 (16.8)	100 (28.8)	< 0.001	1.97 (1.45, 2.66)
BMI ≥ 30 kg/m^2^	401 (44.1)	200 (61.5)	< 0.001	2.07 (1.58, 2.70)
HDP with severe features^a^	105 (10.7)	58 (16.7)	0.005	1.71 (1.17, 2.49)
Thrombocytopenia^b^	15 (1.5)	12 (3.5)	0.02	2.66 (1.17, 6.06)
Cervical Ripening	583 (59.5)	246 (70.9)	0.001	1.63 (1.23, 2.16)

*aOR* Adjusted Odds Ratio, *CI* Confidence Interval, *BMI* Body Mass Index, *HDP* Hypertensive Disorders of Pregnancy

Data are presented as N (%)

Adjusted for maternal age, BMI, severe HDP, thrombocytopenia, and need for cervical ripening

Magnesium sulfate and antihypertensive treatment were highly correlated with severe HDP and therefore excluded from the final model to avoid multicollinearity

^a^Defined as any of the following: severe preeclampsia, eclampsia, HELLP syndrome or superimposed preeclampsia with severe features

^b^Defined as platelet count < 100 × 10⁹/L

**Fig. 2:**
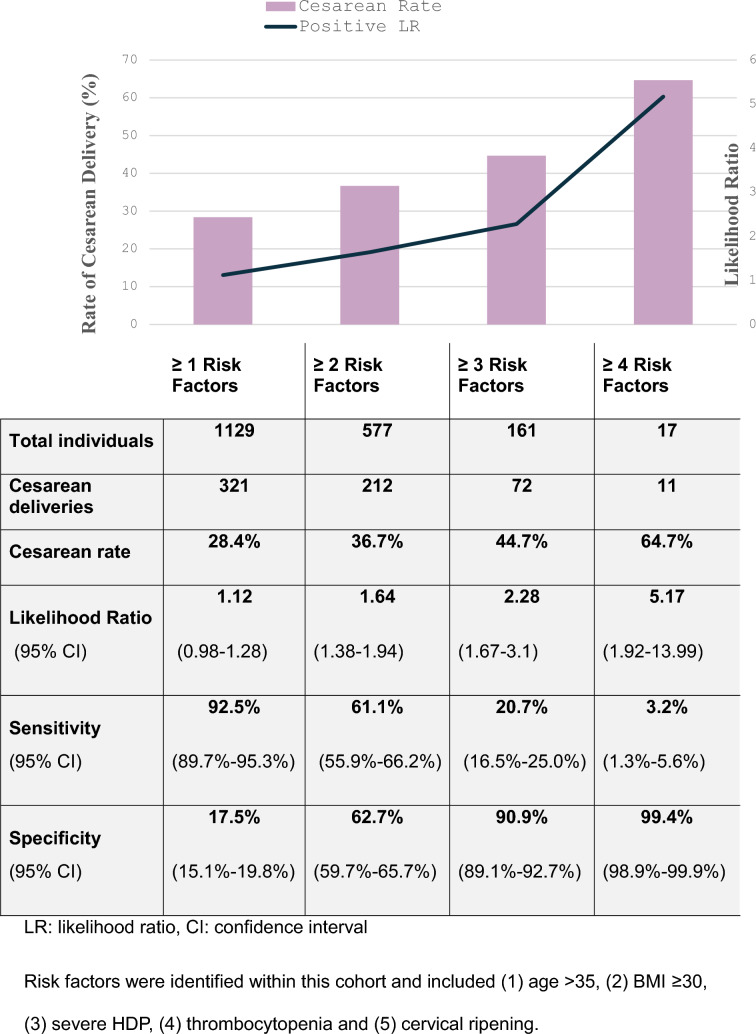
Cesarean delivery rate and likelihood ratios according to number of risk factors for cesarean delivery. Cesarean delivery rates and diagnostic performance (likelihood ratios, sensitivity, specificity) across thresholds of cumulative risk factors (≥ 1, ≥ 2, ≥ 3, ≥ 4). Risk factors included: age > 35 years, BMI ≥ 30 kg/m^2^, HDP with severe features, thrombocytopenia, and need for cervical ripening. LR: likelihood ratio, CI: confidence interval. Risk factors were identified within this cohort and included (1) age > 35, (2) BMI ≥ 30, (3) severe HDP, (4) thrombocytopenia and (5) cervical ripening

Risk stratification by the cumulative number of identified risk factors demonstrated a progressive increase in CD rates: 28.4% with ≥ 1 factor, 36.7% with ≥ 2, 44.7% with ≥ 3, and 64.7% with ≥ 4. The presence of ≥ 4 factors yielded a positive LR of 5.17 (95% CI 1.92–13.99) (Fig. 2).

## Discussion

In this cohort of term nulliparous individuals with HDP undergoing IOL, one in four required CD. Five pre-induction risk factors – age > 35 years, body mass index ≥ 30 kg/m^2^, severe disease, thrombocytopenia, and need for cervical ripening—were independently associated with unplanned cesarean risk, which increased stepwise from 28% with one factor to 65% when four or more were present.

In the general obstetric population, multiple studies have identified maternal and intrapartum characteristics associated with CD following IOL. Among the principal risk factors, higher maternal BMI, advanced maternal age, shorter stature, and low Bishop score on admission have consistently been linked with lower induction success and higher cesarean rates [11–13]. Similar associations between maternal characteristics and mode of delivery have also been reported in studies of trial of labor among nulliparous individuals [14]. Reported CD rates among nulliparous individuals induced at term are approximately 30% [13]. In our cohort of term nulliparous individuals with HDP, the overall CD rate was 26%, comparable to these population-based findings. Maternal obesity and age above 35 years were among the strongest independent predictors, consistent with prior studies in the general obstetric population [11, 13, 15].

Our findings extend and refine prior efforts to predict mode of delivery in individuals with HDP IOL. Existing models have typically examined broad obstetric populations or combined parity and gestational age groups, limiting their applicability to term nulliparous individuals—a group where clinical counseling about delivery outcomes remains particularly complex.

Beninati et al. [10] developed a prediction model for vaginal birth after induction in individuals with HDP using a large U.S. cohort that included both nulliparous and multiparous individuals delivering at ≥ 34 weeks of gestation (*N* = 1,357). Their model incorporated eight predictors—such as maternal age, BMI, gestational age, cervical exam findings, magnesium sulfate exposure, and prior obstetric history—and demonstrated good discrimination (AUC 0.76). However, markers of disease severity, such as thrombocytopenia or elevated liver enzymes, were not predictive in their model, likely due to their low prevalence (< 6%) in the cohort. Furthermore, their model was not designed specifically for nulliparous individuals at term. Our study, by contrast, is focused exclusively on term nulliparous individuals with HDP**,** a well-defined, high-risk population for whom decision-making around induction is highly consequential. We identified five independent pre-induction predictors of unplanned cesarean delivery: maternal age > 35 years, BMI ≥ 30 kg/m^2^, severe HDP, thrombocytopenia, and the need for cervical ripening**.** Importantly, our inclusion of disease severity markers—which were significantly associated with cesarean delivery—adds granularity and clinical relevance to prior work, particularly in light of their exclusion or non-significance in earlier models.

Similarly, the van der Tuuk et al. [16] study, a secondary analysis of the HYPITAT trial [7] evaluated predictors of CD in individuals with gestational hypertension or mild preeclampsia at term. While their models achieved good discrimination (AUC 0.74–0.80), the study excluded individuals with severe disease and had a relatively low cesarean rate (17%). Their findings may thus have limited generalizability to current clinical practice, where induction is frequently undertaken in more complex HDP cases. In contrast, our study includes a broader spectrum of hypertensive severity—including preeclampsia with severe features, allowing for a more representative and pragmatic risk assessment.

### Clinical and research implications

This study offers a practical, cohort-based framework for anticipating CD risk in term nulliparous individuals with HDP undergoing labor induction. By relying solely on routinely available antepartum data, the model enables providers to engage in individualized, data-driven counseling at the bedside**.** This can help align patient expectations, reduce decisional conflict, and promote shared decision-making, especially when discussing the potential benefits and risks of induction versus planned CD in medically indicated contexts.

Although the model is based on a large, well-characterized cohort and relies on routinely available variables, future studies should aim to externally validate this model in diverse populations and settings and evaluate whether implementation improves patient counseling, satisfaction, and delivery outcomes.

### Strengths and limitations

The strengths of our study include a large, well-characterized cohort of term nulliparous individuals with HDP, drawn from a high-volume tertiary care center with a standardized electronic medical record and perinatal database. By focusing on a clearly defined population, we provide precise and clinically relevant estimates applicable to a common but high-risk clinical scenario. The study uses uniform, routinely available pre-induction variables, enhancing the model’s feasibility for real-time clinical application. Importantly, the inclusion of disease-severity markers—such as thrombocytopenia and severe HDP—adds a novel dimension that strengthens the model’s relevance for risk stratification beyond traditional obstetric predictors. The stepwise design of the risk model allows for practical bedside use without requiring digital tools or detailed cervical metrics.

The study is not without limitations. First, this was a retrospective, single-center study conducted in a tertiary referral hospital, which may introduce referral bias and reflect local management protocols. The patient population and clinical decision-making patterns in such a setting may differ from those in community or lower-acuity centers, potentially affecting both baseline risk and cesarean delivery thresholds. These factors may limit the generalizability of our findings to other populations and practice environments.

In addition, the use of stepwise variable selection may introduce model instability and limit reproducibility, as it is sensitive to the specific dataset. Alternative approaches, such as clinically informed forced-entry models or penalized regression techniques, may provide more robust estimates and should be explored in future studies. Notably, the predictors identified in our model are consistent with previously established clinical risk factors, supporting the face validity of the model despite the use of stepwise selection.

Although the model demonstrated acceptable discrimination, we did not formally assess calibration or perform internal validation. Therefore, the potential for overfitting cannot be excluded, and the model’s performance should be interpreted with caution. Future studies should include both internal and external validation to confirm its reliability before clinical application**.**

The LR observed at higher risk thresholds, particularly ≥ 4 risk factors, should be interpreted with caution, as small numbers in these strata result in wide confidence intervals and limited precision. These estimates are therefore best viewed as reflecting a general trend of increasing risk rather than definitive thresholds for bedside clinical decision-making.

Although statistically significant differences were observed in birth weight and 5-min Apgar score between groups, the absolute differences were small and likely of limited clinical relevance.

Lastly, cervical status was captured using a proxy variable (need for cervical ripening), as detailed Bishop score components were not uniformly available, which may limit direct comparison with prior models. This measure may also reflect provider judgment and institutional practice patterns, introducing potential variability. Because cervical ripening is typically performed when the cervix is unfavorable (e.g., low Bishop score), this variable likely serves as a pragmatic surrogate for cervical status in retrospective datasets. However, it is not equivalent to a standardized cervical assessment, and this limitation should be considered when interpreting the model and its generalizability.

## Conclusions

Among term nulliparous individuals with HDP undergoing IOL, we identified five routinely available pre-induction factors: advanced maternal age, obesity, severe HDP, thrombocytopenia, and the need for cervical ripening that were independently associated with increased risk of unplanned CD. This simple, pragmatic model enables personalized risk stratification at the bedside and may enhance clinical counseling, decision-making, and patient preparation. By incorporating both traditional obstetric variables and markers of disease severity, our model provides a more nuanced estimate of CD risk in a high-risk population.

Prospective validation in diverse settings and evaluation of its impact on counseling quality and clinical outcomes are warranted before broader implementation.

## Data Availability

The data that support the findings of this study are not openly available and are available from the corresponding author upon reasonable request.
